# Deletion of *NGG1* in a recombinant *Saccharomyces cerevisiae* improved xylose utilization and affected transcription of genes related to amino acid metabolism

**DOI:** 10.3389/fmicb.2022.960114

**Published:** 2022-09-08

**Authors:** Cheng Cheng, Wei-Bin Wang, Meng-Lin Sun, Rui-Qi Tang, Long Bai, Hal S. Alper, Xin-Qing Zhao

**Affiliations:** ^1^State Key Laboratory of Microbial Metabolism, School of Life Sciences and Biotechnology, Shanghai Jiao Tong University, Shanghai, China; ^2^School of Life Sciences, Hefei Normal University, Hefei, China; ^3^McKetta Department of Chemical Engineering, The University of Texas at Austin, Austin, TX, United States

**Keywords:** *Saccharomyces cerevisiae*, xylose utilization, *NGG1*, transcriptome analysis, amino acid metabolism

## Abstract

Production of biofuels and biochemicals from xylose using yeast cell factory is of great interest for lignocellulosic biorefinery. Our previous studies revealed that a natural yeast isolate *Saccharomyces cerevisiae* YB-2625 has superior xylose-fermenting ability. Through integrative omics analysis, *NGG1*, which encodes a transcription regulator as well as a subunit of chromatin modifying histone acetyltransferase complexes was revealed to regulate xylose metabolism. Deletion of *NGG1* in *S. cerevisiae* YRH396h, which is the haploid version of the recombinant yeast using *S. cerevisiae* YB-2625 as the host strain, improved xylose consumption by 28.6%. Comparative transcriptome analysis revealed that *NGG1* deletion down-regulated genes related to mitochondrial function, TCA cycle, ATP biosynthesis, respiration, as well as NADH generation. In addition, the *NGG1* deletion mutant also showed transcriptional changes in amino acid biosynthesis genes. Further analysis of intracellular amino acid content confirmed the effect of *NGG1* on amino acid accumulation during xylose utilization. Our results indicated that *NGG1* is one of the core nodes for coordinated regulation of carbon and nitrogen metabolism in the recombinant *S. cerevisiae.* This work reveals novel function of Ngg1p in yeast metabolism and provides basis for developing robust yeast strains to produce ethanol and biochemicals using lignocellulosic biomass.

## Introduction

Xylose is an abundant and renewable sugar, which ranks second only to glucose in nature. Efficient xylose utilization is of great importance for biorefinery of lignocellulosic biomass. Budding yeast *Saccharomyces cerevisiae* has been widely used to produce biofuels and biochemicals using renewable biomass, especially lignocellulosic feedstocks (Baptista et al., 2021). Development of yeast strains with improved xylose utilization performance is critical for the applications of lignocellulosic biorefinery (Lee J. W. et al., 2021). Great efforts have been focused on metabolic engineering and evolutionary engineering to improve xylose utilization of recombinant *S. cerevisiae* strains (Sandberg et al., 2019; Cunha et al., 2020; Sun et al., 2022). However, the performance of recombinant xylose-assimilating strains still cannot satisfy the requirement of industrial production. Exploration of more native xylose-fermenting *S. cerevisiae* isolates and utilization of robust industrial yeast strains are promising strategies (Wenger et al., 2010; Romaní et al., 2015; Cheng et al., 2018; Cunha et al., 2019). To enable rational engineering of these superior chassis strains, it is of great importance to further explore how xylose metabolism is regulated in yeast cells.

Currently, some key factors regulating xylose utilization in the recombinant xylose utilizing strain have been explored, such as *ASK10*, *PHO13*, *GCR2*, *HOG1*, and *IRA2* (Hou et al., 2016; Sun and Jin, 2021; Shin et al., 2021). It is estimated that xylose metabolism of yeast cells is under synergistic regulation by multiple factors (Michael et al., 2016). Generally, the effects achieved by manipulation of single transcription factors (TFs) were not satisfactory, and comprehensive regulation of the metabolic network is always required (Myers et al., 2019). Therefore, it is very important to explore global regulators and the innate regulatory mechanisms of xylose metabolism in the xylose-utilizing *S. cerevisiae* (Endalur Gopinarayanan and Nair, 2019; Sun and Jin, 2021).

In eukaryotes, the ATP-dependent chromosome remodeling complex and histone modification enzymes are both involved in the regulation of DNA accessibility, which affects transcriptional state (Hargreaves and Crabtree, 2011). For instance, acetylation of histones H3 and H4, and recruitment of Spt-Ada-Gcn5-Acetyltransferase (SAGA) complex and NuA4 complex to promoters are critical parts of transcriptional regulation (Galdieri et al., 2014). Deletion of genes *SWI3*, *RSC1*, *SPT10*, *UME6*, or *NGG1* encoding components of chromatin remodeling complexes, increased static ethanol fermentation of *S. cerevisiae* BY4743 derivative strain (Fang et al., 2020). On the other hand, the Tup1p-Cyc8p complex plays a role in recruiting histone deacetylase to the targeted genes. It was reported that a mutated *CYC8* was found in an adapted yeast with improved xylose-fermenting ability under glucose repression. When introducing the mutant gene *mCYC8* to the control strain, transcriptomic level of multiple genes was changed, including hexose transport and maltose metabolism (Nijland et al., 2017). Recently, it was identified that the SWI/SNF chromatin remodeling was responsible for the improved growth after switching the carbon source from glucose to xylose for yeast cells (Li et al., 2021). It will be interesting to explore in-depth mechanisms on the roles of chromosome remodeling and histone modification factors for xylose utilization.

In our previous study, a natural yeast isolate *S. cerevisiae* YB-2625 was shown to have superior xylose consumption efficiency when compared with the model strain *S. cerevisiae* S288c (Cheng et al., 2018) as well as other yeast strains of *S. cerevisiae* (unpublished data). Comparative transcriptomic analysis has been performed to reveal the major mechanisms for naturally xylose-fermenting ability of YB-2625 (Cheng et al., 2018). However, global regulatory factors involved in xylose metabolism of this unique *S. cerevisiae* remained unexplored.

In this study, we performed integrative analyses of genomic sequence and transcriptome data, and further focused on *NGG1*, which encodes a transcription activator and plays an important role in chromatin modification, more specifically, acetylation of histone H3 and H2B (Lee et al., 2011). Previous studies reported that Ngg1p was a negative regulator of Gal4p, which participates in glucose repression (Brandl et al., 1993). In a recent report, loss of *NGG1* increased ethanol production during static ethanol fermentation (Fang et al., 2020). It has been confirmed that *NGG1* was associated with the metabolism of non-fermentable substrate: under the glucose-restricted condition in continuous culture, Ngg1p (Ada3p) was shown to be essential for the acetylation of Spt7p and Sgf73p, which are components of the SAGA transcriptional coactivator complex, to response to the non-fermentable substrates such as acetate (Cai et al., 2011). Despite the above-mentioned reports, so far, no studies have been focused on the influence of Ngg1p on xylose metabolism.

The aim of the study is to investigate the global regulation network of *NGG1* under the condition of xylose fermentation in the recombinant *S. cerevisiae*. Our results provide novel insights in regulation of xylose utilization and nitrogen metabolism in recombinant *S. cerevisiae*, and benefit in-depth understanding on the role of *NGG1* in yeast metabolism.

## Materials and methods

### Strains and culture conditions

*Escherichia coli* DH5α was used for plasmid maintenance and propagation, and cultured in Luria–Bertani (LB) medium with 100 μg/mL Ampicillin added when necessary. Yeast strains used in this study are listed in Supplementary Table 1. YPD medium consisting of 10 g/L yeast extract, 20 g/L peptone, and 20 g/L glucose was used for yeast cell cultivation, and 20 g/L agar was added to the medium to prepare solid medium. To screen transformants, 300 μg/mL hygromycin B was supplemented in medium. YPX40 medium composed of 4 g/L yeast exact, 3 g/L peptone, and 40 g/L xylose was used for xylose fermentation. After seed culture in YPD medium, the yeast cells were inoculated at 10% (v/v) into 250-mL flasks containing 100 mL YPX40, and fermentation was performed at 30°C, 150 rpm under micro-aerobic condition.

### Sporulation of YRH396 and growth ability determination

To obtain haploid strain from YRH396, the yeast strain was precultured in YPD plates at 30°C for three times, which was then transferred to McClary plate (0.1% glucose, 0.18% potassium chloride, 0.82% sodium acetate, 0.25% yeast extract, 2% agar). After cultivating at 30°C for 7 days, the cells were washed by 1-mL physiological saline (0.9% NaCl). The yeast cells were then harvested by centrifugation at 2,400 × *g* for 5 min, and the pellets were hydrolyzed by 1 mL of 20 mg/L snailase at 37°C for 2 h, followed by treatment at 58°C for 8 min. The cell suspension was then immediately spread on YPD plates and incubated at 30°C until colony formation. Further, mating type of the haploids was identified by PCR analysis as previously reported (Huxley et al., 1990). The cells of varied haploids were cultivated in 48 deep-well baffled plates by 10% inoculation size in 1 mL YPX40 or YPD80X40 medium for culture at 220 rpm and 30°C for 96 h, then cell growth was determined by checking the optical density at 600 nm.

### Stress tolerance assay of varied yeast strains

Stress tolerance of yeast strains was evaluated by spot assay. Yeast cells were cultured in YPD medium at 30°C for 24 h, and then transferred to fresh YPD medium until stationary phase. OD_600_ of the broth was adjusted to the same level with distilled water, and 2.0 μL of the ten-fold diluted suspensions of each strain was spotted on solid YPD medium supplemented with 5 mM hydrogen peroxide, 83.3 mM acetic acid, or 10% ethanol (Chen et al., 2022). YPD medium without inhibitors addition were regarded as the control. The plates were incubated at 30°C for 2 days, while solid YPD medium incubated at 40°C were used for evaluating the thermal tolerance of the different yeast strains.

### Construction of gene deletion mutants

Disruption of *NGG1* in YRH396h was performed using homologous recombination method. DNA fragments were amplified by PCR using plasmid pRS41H as templet with primer pairs Δ*ngg1*-F/R (Supplementary Table 2). The obtained cassettes contained HphMX marker sequence and 40 bp homologous region of the targeted genes. Deletion mutants were constructed by transforming the cassettes into YRH396h using the lithium acetate method (Gietz and Schiestl, 2007). The transformants were verified by PCR analysis with primer pairs NGG1-F/hph-in-R after extracting genomic DNA. The mutant lacking *NGG1* was named as YRH396h-*ngg1*Δ.

### Western blot analysis

Samples were taken at 48 h during xylose fermentation with 40 g/L xylose. Cell pellets collected by centrifugation at 10,000 × *g* for 10 min were washed with sterilized distilled water for twice. Whole cell lysate was obtained by vertexing the cells with 0.5-mm glass beads (Couttas et al., 2012). The content of cell protein was determined using BCA protein kit according to the manufacturer’s instructions (Beyotime Institute of Biotechnology, Jiangsu, China). Totally, 20 μg of whole cell lysate of YRH396h and YRH396h-*ngg1*Δ was used for Western blot analysis. The pan acetylation profile in the yeast cells was performed with anti-acetyl lysine antibody (PTM-101; 1:1000 dilution) as primary antibody and peroxidase conjugated Goat anti-Mouse IgG (H + L) as 2nd antibody (1:5000 dilution). The blots were detected by chemiluminescence.

### Comparative transcriptome analysis and real-time quantitative PCR analysis

For comparative transcriptome analysis, strains of YRH396h and YRH396h-*ngg1*Δ were cultured in YPX40 medium, and yeast cell grown at exponential phase (48 h) was harvested by centrifugation at 8,000 × *g* for 5 min. Total RNA was extracted using the Spin Column Plant total RNA Purification Kit (Sangon, Shanghai, China). RNA quality and quantity as well as insert size of the libraries were determined by Agilent 2100 Bioanalyzer. Sequencing data were produced by Illumina Hiseq 4000 and analyzed by Beijing novogene Technology Co., Ltd. After differential expression gene analysis with the software of HTSeq, the quality and reproducibility of the data were assessed by calculation of Pearson Correlation Coefficient. *R*^2^ value of the two biological replicates was more than 0.925, suggesting that the results were reliable. Based on the differentially expressed genes (Log_2_ ratio ≥ 0.5 or ≤ −0.5, FDR ≤ 0.001), in-depth analysis including Gene Ontology (GO) enrichment^1^ and enriched TFs regulating the obviously changed genes^2^ were further conducted.

RT-qPCR experiments were conducted following the manufacturer’s instruction of SYBR Green dye (PrimeScript RT reagent Kit With gDNA Eraser), and *ALG9* was selected as the house-keeping gene (Teste et al., 2009). Relative expression level of the genes was calculated by the 2^–ΔΔ^ ^Ct^ method (Livak and Schmittgen, 2001). Primers sequences used for RT-qPCR analysis are listed in Supplementary Table 3.

### Enzyme activities assay of XR, XDH, and XK

Enzyme activities assay was measured according to the previous study (Eliasson et al., 2000). The assays were performed in triplicates and the average values were presented.

### Detection of the intracellular amino acid contents

After cultured under the same condition of transcriptomic analysis, yeast cells were collected at 4°C, 3000 × *g* for 5 min, and then washed with sterilized distilled water twice. The pellets were re-suspended to 800 μL in 100 mM hydrochloric acid. After cell lysis and centrifugation at 4°C, 12000 × *g* for 5 min, and 400 μL supernatant was obtained and mixed sufficiently with 200 μL 16.7% (w/v) sulfosalicylic acid. The mixtures were incubated at 4°C for 1 h. Subsequently, the supernatant was collected under the condition of 4°C, 15000 × *g* for 30 min, and then its pH value was adjusted to 1.7–2.2 by the addition of NaOH (Zhang et al., 2019). After filtration, the quantification of intracellular amino acids of yeast cells was carried out by amino acid analyzer (HITACHI L-8900, Japan).

### High-performance liquid chromatography analysis

All collected samples were firstly centrifuged at 10,000 × *g* for 2 min, and then analyzed by HPLC (Waters e2695, MA, United States) equipped with the Aminex HPX-87H column (300 mm × 7.8 mm, Bio-Rad, Hercules, CA) and the refractive index detector (Waters 2414 RID, MA, United States) following the previously developed protocol (Wang et al., 2013).

### Statistical analysis

All quantitative data were expressed as the mean value with corresponding standard deviation (SD) obtained from three independent experiments. The statistical analysis showed in amino acids detection section was performed using Student’s *t*-test at the significance of *P* < 0.05 and *P* < 0.01, respectively.

## Results and discussion

### Analysis of mutation sites in the *NGG1* sequence of *Saccharomyces cerevisiae* YB-2625

**FIGURE 1:**
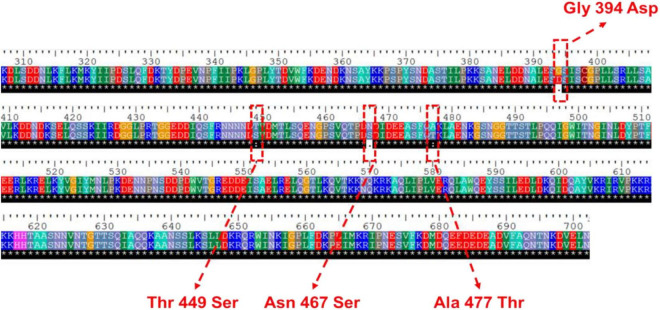
Variations in amino acid sequence of Ngg1p in *S. cerevisiae* YB-2625 compared to *S. cerevisiae* S288c.

In our previous study, comparative transcriptomic analysis between *S. cerevisiae* YB-2625 and the model yeast strain S288c was performed at the mixed-sugar (xylose and glucose) utilization stage (XG stage) and xylose-utilization stage (X stage) (Cheng et al., 2018). Notably, *NGG1* showed lower transcription level by −0.67 and −1.11-fold at XG and X stage, respectively. In the protein sequence of Ngg1p, amino acids located in 364-702 sites act as the carboxyl-terminal domain, which is responsible for binding to Ada2p and Gcn5p (Horiuchi et al., 1995). There are four non-synonymous mutations (Gly349Asp, Thr449Ser, Asn467Ser, and Ala477Thr) in the genome sequence of YB-2625 compared to S288c (Figure 1). Except for Gly349Asp, the other three mutations were all located in carboxyl-terminal domain of Ngg1p, which might affect its binding to acetyltransferase Gcn5p and then have an influence on histone acetylation. Ngg1p is required for histone acetyltransferase (HAT) activity, lysine specificity, and maintenance of SAGA structure (Balasubramanian et al., 2002). Furthermore, Ada2p and Ngg1p cooperate with Gcn5p to regulate the acetylation of non-histone substrates, and Ngg1p is a global regulator of acetylation (Rössl et al., 2019). As shown in Supplementary Figure 1, protein-acetylated level in the cells with *NGG1* deletion was indeed lower than that of the control, which suggests that *NGG1* deficiency affects global acetylation state of the yeast.

### Effects of *NGG1* deletion on stress tolerance and xylose-fermentation efficiency

#### Isolation of the haploid strain *Saccharomyces cerevisiae* YRH396h for further evaluation

Considering the difficulty in genetic manipulation of diploid yeast, we firstly obtained haploid derivatives of the xylose-fermenting yeast YRH396, which was constructed using *S. cerevisiae* YB-2625 as the parent strain (Hector et al., 2011). Sixteen haploid isolates were obtained. Previous studies showed that haploid strains with superior desired phenotypes may be obtained from their diploid or triploid parents (Kim et al., 2017). We then evaluated xylose and mixed-sugar fermentation performance using the obtained haploids. As shown in Supplementary Figure 2, growth ability of the haploid strains was different, with OD_600_ varying from 5.4 to 10.6 in mixed-sugar and 4.2 to 6.1 in xylose after cultivated for 96 h. Remarkably, the most robust haploids grown in xylose (No. 2, 8, 14) showed weakened growth ability in the presence of mixed sugar, which was not suitable for bioethanol production. After comprehensive consideration of the growth ability in mixed sugar and xylose, a haploid strain No.3 was selected for further genetic modification, which was named YRH396h.

#### Varied stress tolerance ability of the *NGG1* deletion mutant

To explore the function of *NGG1* in yeast cells, a *NGG1* gene deletion strain was constructed from the xylose-fermenting haploid yeast strain YRH396h. Based on the important characteristic requirements of yeasts for lignocellulosic ethanol production, stress tolerance and xylose metabolism abilities of the mutant were examined.

**FIGURE 2:**
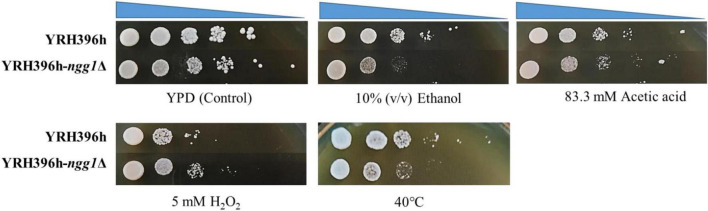
Stress tolerance of the *NGG1* deletion mutant of *S cerevisiae* YRH396h based on the genetic background of *S. cerevisiae* YB-2625. Stress tolerance of the yeast strains was evaluated by spot assay. The initial OD_600_ of all yeast strains was adjusted to 1.0, and yeast cells were diluted by ten-fold serially. YPD agar medium without inhibitors was used as a control medium. Stress tolerance of the yeast strains was tested in YPD agar medium supplemented with 10% (v/v) ethanol, 83.3 mM acetic acid, or 5 mM H_2_O_2_, respectively. Except for thermal tolerance test at 42°C, the yeast strains were all cultivated at 30°C.

As shown in Figure 2, YRH396h-*ngg1*Δ showed weaker growth ability compared to the control strain without the *NGG1* deletion under stresses of acetic acid, high temperature, and ethanol. In contrast, improved cell growth of YRH396h-*ngg1*Δ was observed when 5 mM H_2_O_2_ was supplemented. Enhanced oxidative stress tolerance was confirmed to be beneficial for xylose utilization ability of *S. cerevisiae* in our previous study (Cheng et al., 2018). The results here also indicate that the absence of *NGG1* impacts multiple stress responses of yeast cells. The previous report revealed that the *NGG1* deletion strain derived from yeast strain BP1 was temperature-sensitive, and the mutant showed no growth at 37°C (Piña et al., 1993). However, the repression of growth by *NGG1* absence in YRH396h in the current study was not significant, which suggested that *NGG1* deletion has different impact on thermotolerance of yeast strains with diverse genetic background. Our results agree with the previous report that the influence of genetic manipulation was dependent on yeast strains (Cunha et al., 2015).

According to our previous studies, deficiency of histone H3 acetyltransferase, Rtt109p, enhanced acetic acid resistance of *S. cerevisiae* by regulating the expression level of stress responsive genes (Cheng et al., 2016). Additionally, we also found that overexpression of *SET5*, which encodes a methyltransferase of histone H4, benefits stress tolerance of yeast cells (Zhang et al., 2015). Combining with the previous report, our results further point out the importance of histone modification in regulation of environmental stress response of *S. cerevisiae*.

#### Enhanced xylose utilization and ethanol fermentation by *NGG1* deletion

**FIGURE 3:**
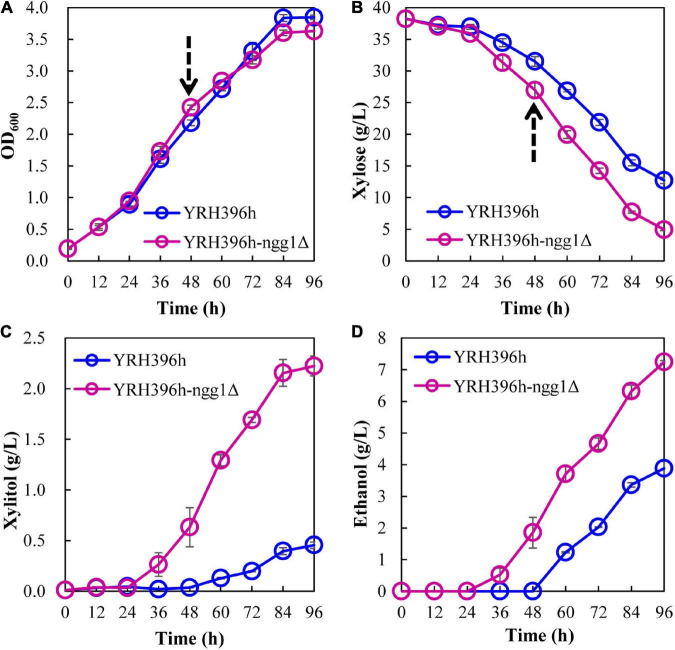
Effect of *NGG1* deletion on xylose fermentation of *S. cerevisiae* YRH396h. Batch fermentation was performed in 100 mL medium containing 4 g/L yeast extract, 3 g/L peptone, and 40 g/L xylose in 250 mL Erlenmeyer flasks with the initial OD_600_ of ∼0.2 shaking at 150 rpm and 30°C under micro-aerobic condition. **(A)** Growth ability, **(B)** Xylose consumption, **(C,D)** Xylitol and ethanol generation. The arrows at 48 h indicate the time points of sample collection for comparative transcriptomic analysis.

Deletion of *NGG1* in laboratory *S. cerevisiae* BY4741 resulted in significant impairment of yeast growth in synthetic (SC-Ura) medium (Wu et al., 2017). In our study, no obvious effect in cell growth was found when fermenting in 40 g/L xylose by knocking out *NGG1* in YRH396h (Figure 3), suggesting that other factors in the genome of *S. cerevisiae* YB-2625 may function together with Ngg1p for the growth regulation. As can be seen, deletion of *NGG1* facilitated higher xylose consumption of YRH396h. After fermenting for 96 h, the *NGG1* deletion mutant consumed 35.1 g/L xylose and generated 7.24 g/L ethanol, which achieved 28.6% and 86.6% increase, respectively, than that of the control strain. In the meanwhile, xylitol production dramatically increased from 0.46 g/L in the control strain to 2.22 g/L in YRH396h-*ngg1*Δ. These results demonstrated here that deletion of *NGG1* exerts a positive effect on both xylose metabolism and ethanol production. Ngg1p is the component of SAGA, which are the HAT complexes. Our results here are consistent with the recent study that disruption of *GCN5*, which encodes another component of SAGA, benefits xylose consumption (Tan et al., 2021).

### Changed transcriptomic profile by *NGG1* deletion during xylose metabolism

#### Overview of the transcriptomic analysis data

To validate the RNA-seq analysis data, transcriptional level of five genes was confirmed by RT-qPCR analysis. Among these genes, *XKS1* is the key gene related to xylose metabolism, while *SOD1*, *PRX1*, and *CTT1* encode enzymes for oxidative stress response. Additionally, the expression level of *ASK10*, a stress response regulator, was also tested. As shown in Supplementary Table 4, the results of RT-qPCR and RNA-seq analysis are consistent, indicating the reliability of the transcriptomic data. Comparative transcriptomic analysis showed that 720 differentially expressed genes were up-regulated and 863 genes were down-regulated.

#### Transcription factor enrichment analysis of the transcriptomic data

Top 20 enriched TFs regulating the significantly changed genes were listed in Supplementary Figure 3. Among the top five enriched TFs, Ace2p and Sfp1p are known to benefit ethanol production under stress conditions (Chen et al., 2016), but their effect on xylose fermentation has not been reported. Besides that *MSN2* is a stress-responsive transcriptional activator for various stress conditions. *YAP1* is a key gene for oxidative stress response, and it was involved in xylose metabolism regulation regardless of host strain or xylose-fermenting pathways (Feng and Zhao, 2013a). As reported, changed expression level of oxidative stress related TFs was also found in yeast strains with mutated NADH-preferring XR, which showed improved xylose utilization (Xie et al., 2020). In our current study, the significant enrichment of Msn2p/Msn4p, Yap1p, and Ste12p indicated that stress response is related to improved xylose utilization by *NGG1* deletion. This finding agrees with a recent report that deletion of the general stress response regulator *MSN4* increased cell fitness of the recombinant strain grown in xylose (Li et al., 2021).

Among the top 20 TFs, four TFs were required for general transcriptional regulation. For example, Fkh1p is one of the key TFs controlling transcription elongation. Rap1p and Abf1p are both DNA-binding proteins related to transcriptional activation. Tup1p is a general repressor of transcription, forming a complex with Cyc8p and involving in the establishment of repressive chromatin structure through interactions with histones H3 and H4. The enrichment of these TFs suggested that *NGG1* deletion affected the process of genes transcription, which is consistent with the role of *NGG1* as a transcription regulator. Additionally, deletion of *NGG1* caused differential expression of multiple regulating factors encoding genes, such as *ASK10*, *ISU1*, *IRA2*, *CAT8*, and *ADR1* (Supplementary Table 5). Manipulation of these TFs was demonstrated to generate significant changes in carbon metabolism behavior of yeast. For instance, improved ethanol production was achieved by disruption of *CAT8* or *ADR1* (Michael et al., 2016). The results here suggested that Ngg1p acts as a global regulator for carbon metabolism in *S. cerevisiae*.

The TF enrichment analysis also revealed enrichment of Gcn4p (Supplementary Figure 3), which is a key TF regulating of amino acid biosynthesis, and was previously proposed to play an important role in the regulation of xylose utilization (Feng and Zhao, 2013a). This implied that the xylose fermentation of YRH396h-*ngg1*Δ might be affected by amino acids metabolism, which promoted us to perform further studies (refer section “Metabolism of amino acids”).

#### Differentially expressed genes related to central carbon metabolism

**FIGURE 4:**
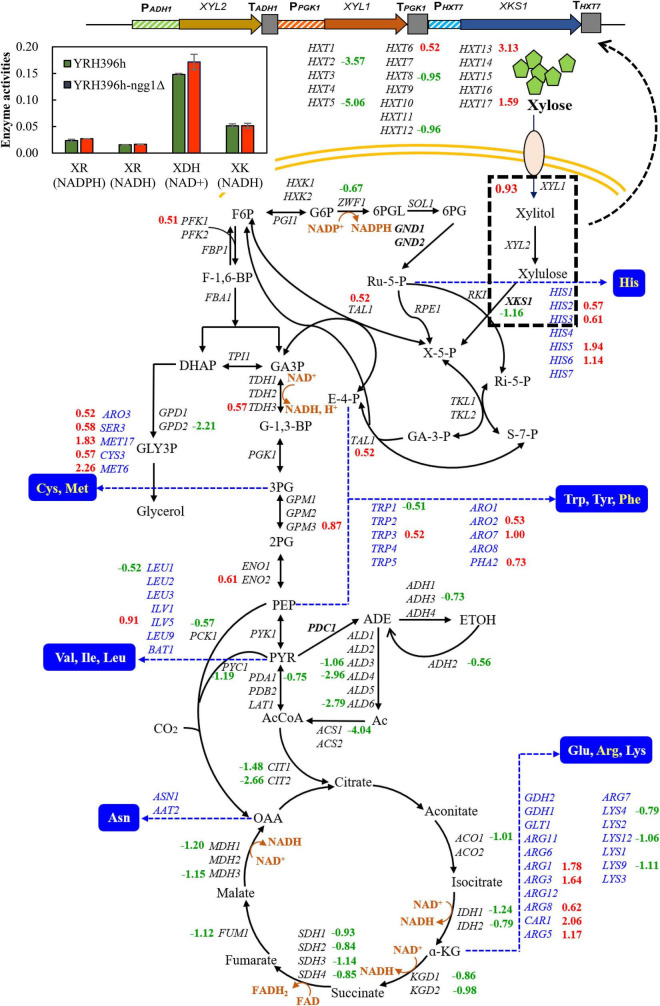
Differentially expressed genes involved in carbohydrate and amino acids metabolism between YRH396h-*ngg1*Δ and YRH396h. Yeast strain with *NGG1* deletion and its parent strain *S. cerevisiae* YRH396h were grown in YPX40 medium consisting of 4 g/L yeast exact, 3 g/L peptone, and 40 g/L xylose, and cells were collected at 48 h for transcriptome analysis. Genes involved in central carbon metabolism and amino acid biosynthesis were presented. The heterogenous xylose metabolism pathway containing *XYL1*, *XYL2*, and *XKS1* as well as relative enzyme activities (XR, XDH, and XK) were also displayed in the upper left panel.

According to the comparison between YRH396h-*ngg1*Δ and YRH396h, a large number of genes involved in central carbon metabolism showed differential transcription levels (Figure 4). In YRH396h-*ngg1*Δ with xylose metabolism pathway consisting of *XYL1*, *XYL2*, and *XKS1*, enhanced transcription of *XYL1* by 0.93-fold, lower transcription level of *XKS1* by −1.16-fold, and no difference in *XYL2* were found. The unbalanced expression of *XYL1*, *XYL2*, and *XKS1* might be a reason for xylitol accumulation in the *NGG1* deletion mutant (Zhu et al., 2021). Therefore, optimized expression of *XYL2* could be a target to decrease the by-product. In YRH396h, xylose-fermenting pathway was introduced exogenously, and expression of *XYL1*, *XYL2*, and *XKS1* was controlled by different promoters including *PGK1p*, *ADH1p*, and *HXT7p* (Hector et al., 2011). Chromatin remodeling complexes may affect gene expression mainly through its effect on promoter activity (Lorch and Kornberg, 2015). In addition, Gcn5-dependent H3K14 acetylation also targeted in the gene coding region and influence transcription elongation (Church and Fleming, 2018). In this study, obviously changed genes *XYL1* and *XKS1* combining with stable expression of the native genes, *PGK1* and *HXT7*, were found. We speculated that *NGG1* deletion might affect transcription elongation of these genes in the yeast cells. Notably, activities of xylose reductase (XR) and xylulose kinase (XK) remained unchanged when deleting *NGG1* (Figure 4). This inconsistency of varied transcription level and enzyme activities suggested that the gene expression may also be affected by other regulatory processes, such as post-transcriptional, mRNA translational, and posttranslational modifications.

Non-oxidative pentose phosphate pathway is a limited step for xylose metabolism of yeast cells. Specially, the expression level of *TAL1* was linearly correlated with xylose fermentation capability (Xu et al., 2016). The enhanced expression of *TAL1* by 0.52-fold was revealed in the *NGG1* deletion mutant compared to YRH396h. It is worth noting that transcription levels of the TCA cycle related genes were generally decreased by *NGG1* deletion. These genes include *ACO1*, *IDH1*, *IDH2*, *KGD1*, *KGD2*, *SDH1-4*, *FUM1*, *MDH1*, *MDH3*, *CIT1*, and *CIT2*. The activity of TCA cycle was closely related to energy maintenance and biomass accumulation. As reported previously, in the engineered xylose-fermenting *S. cerevisiae*, reducing of metabolic flux distributed to TCA cycle and energy maintenance was a crucial strategy for increased ethanol production (Feng and Zhao, 2013b). Intriguingly, multiple genes involved in ATP biosynthesis were obviously down-regulated in the cells of YRH396h-*ngg1*Δ, such as *ATP1*, *ATP3*, *ATP4*, *ATP5*, *ATP7*, *ATP14*, *ATP15*, *ATP17*, *ATP19*, and *ATP20*. The lower transcriptional level of genes involved in energy generation in YRH396h-*ngg1*Δ is consistent with the increased production of ethanol (Figure 3).

More importantly, transcription of the enzyme encoding genes for oxidoreductive reactions in TCA cycle was all decreased (Figure 4). The reactions catalyzed by isocitrate dehydrogenase, alpha-ketoglutarate dehydrogenase and malate dehydrogenase are all NAD^+^-dependent, and thus decreased transcription level of these genes are conducive to NAD^+^ accumulation, which could alleviate cofactor unbalance of xylose metabolism pathway. Coupled with that, down-regulation of *ZWF1*, a glucose-6-phosphate dehydrogenase encoding gene, by −0.67-fold was revealed in this transcriptomic profile. According to the previous study, deletion of *ZWF1* reduced the supply of NADPH and forced NADH utilization by XR, while XDH uses NAD^+^ exclusively. To some extent, the cofactor imbalance was relieved, facilitating the improvement of xylose fermentation (Zha et al., 2014). In this study, we determined activity of NAD^+^-dependent xylitol dehydrogenase (XDH) and found there was no significant increase in XDH activity of the *NGG1* deletion mutant when compared to that of the control strain (Figure 4). In the meanwhile, xylitol production of YRH396h-*ngg1*Δ was higher than that of the control (Figure 3). As reported, moderate respiration metabolism is beneficial for reduced xylitol formation and NADH re-oxidation (Peng et al., 2012). As shown in Supplementary Figure 4, respiration-related genes were generally down-regulated in the cells of YRH396h-*ngg1*Δ. The higher accumulation of xylitol by the *NGG1* deletion mutant indicated that respiratory depression might cause severe cofactor unbalance as reported previously (Zhao et al., 2020) and have a more significant impact on the xylitol generation. In the further study, balance of the cofactor usage between XR and XDH will be explored as an important strategy to decrease xylitol production in the xylose-utilizing mutant.

#### Respiratory and starvation responses

Enriched GO terms were highlighted in Supplementary Figure 4, and enrichment of down-regulated genes mainly occurred in transcription profile of ATP biosynthesis, oxidation-reduction process, etc. (Supplementary Figure 4). Remarkably, besides TCA cycle, several enrichments were involved in mitochondrion function, for instance, electron transport chain, ATPase activity, mitochondrion membrane, and matrix. Our results agree with the previous studies that there is a crosstalk between mitochondria and epigenetics (Matilainen et al., 2017). In the meanwhile, loss of *NGG1* resulted in lower expression of gene encoding acetyl-CoA synthetase (*ACS1*) by −4.04-fold. It will be interesting to further explore whether respiration was affected by disruption of *NGG1*.

As generally known, respiration-dependent ethanol utilization results in reduced ethanol yield during xylose fermentation. Improved ethanol production was achieved by blocking respiration (Jin et al., 2004). Disruption of electron transfer chain by deleting of *COX4*, which encodes a subunit IV of cytochrome c oxidase, was conducive to the accumulation of ethanol (Peng et al., 2012). In the *NGG1* deletion mutant, the expression of genes related to electron transfer chain and Fe/S cluster was down-regulated, for instance, *COX4* was decreased by −0.88-fold. The genetic characteristics were in agreement with higher ethanol production in the *NGG1* deletion mutant.

On the other hand, when xylose-fermenting recombinant yeast was cultivated in xylose, expression of the genes involved in TCA cycle and respiration was significantly increased compared to that when cells assimilate glucose. Even *S. cerevisiae* engineered for xylose metabolism does not recognize xylose as a fermentable carbon source but exhibits a respiratory response (Jin et al., 2004). Respiration was required for yeast cells to achieve sufficient energy to growth on xylose aerobically (Lee S. B. et al., 2021). As reported previously, shifted metabolic state from fermentation to respiration could be achieved by deletion of *SPT10*, *SWI6*, and *ASF1*, which function in altering chromatin structure (Galdieri et al., 2016). In our results, after *NGG1* deletion, the yeast cell tended to down-regulate respiration-related genes. Therefore, strain YRH396h-*ngg1*Δ very likely recognized the non-preferred xylose as fermentable carbon source.

During growth on non-fermentable carbon sources, transcription of *GUT1* and *GUT2* which encode glycerol kinase and glycerol-3-phosphate dehydrogenase, together with *HXK1*, *GLK1*, *FBP1*, and *PCK1* was reported to be enhanced (Matsushika et al., 2014). In contrast, in the transcriptomic profile of YRH396h-*ngg1*Δ compared to YRH396h, no obvious difference in *HXK1* and *GLK1*, and lower expression of *GUT1*, *GUT2*, *FBP1*, and *PCK1* by −1.89, −2.59, −2.45, and −0.57-fold was found. Additionally, for *S. cerevisiae*, *ATO1*, *ATO2*, and *ATO3* are indicators of cell starvation. Under the xylose-fermenting condition, genes *ATO1-3* were generally up-regulated (Salusjärvi et al., 2006). However, in this study, the opposite trend was revealed: the expression of *ATO2* and *ATO3* was significantly down-regulated by −3.69 and −4.21-fold in YRH396h-*ngg1*Δ than that in the control strain. Our results indicated that relief of starvation state of yeast cells may be achieved by *NGG1* deletion during xylose fermentation. Combinatorial down-regulation of genes involved in respiratory response, growth on non-fermentable carbon sources and cell starvation further confirmed our conjecture that *NGG1* deficiency may be beneficial for recognizing xylose as fermentable carbon source by *S. cerevisiae* strain YRH396.

#### Regulation of intracellular iron of yeast

For the *NGG1* deletion mutant, lower transcription levels of *FIT3* and *FRE3* by −1.44 and −1.29-fold were observed when compared to the control. Both genes participate in iron transportation. Combined with other down-regulated genes for ion transport and metal ion binding as shown in GO analysis (Supplementary Figure 4), it indicated that Ngg1p might participate in iron homeostasis. The activity of XR isolated from *Kluyveromyces marxianus* was completely inhibited by Fe^3+^ (Zhang et al., 2011). In this study, higher expression level of *XYL1* by 0.93-fold indicated that XR derived from *Scheffersomyces stipitis* was also influenced by the intracellular concentration of iron. The genes involved in metal homeostasis are new targets for enhanced xylose metabolism in engineered yeast strains with xylose isomerase (XI) pathway for the reason that XI is a metalloenzyme (Sato et al., 2016; Palermo et al., 2021). Interestingly, besides engineered strains with XI pathway, deletion of *ISU1*, which encodes a Fe-S cluster scaffold protein, had positive effects on xylose metabolism of yeast strains endowed XR-XDH pathway (Sato et al., 2016; Osiro et al., 2019). Down-regulation of *ISU1* (−0.83) was found from our transcriptomic data, which might contribute to better xylose utilization of *NGG1* deletion mutant. Meanwhile, YRH396h-*ngg1*Δ showed decreased expression of *CCC1*, *FRE6*, and *HMX1* by −0.63, −0.73 and −0.94-fold, respectively. Deletion of these iron homeostasis-related genes was conducive to xylose metabolism and ethanol production by yeast cells with XI pathway (Palermo et al., 2021). It suggested that metal homeostasis had a broader relation with the optimization of other metal-dependent enzymes or cell metabolism (Osiro et al., 2019).

Regulation of iron in mitochondrion is crucial for Fe/S cluster assembly which participates in electron transport chain. However, free iron is also a substrate of the Fenton reaction to enhance ROS production (Levi and Rovida, 2009). According to the recent study, repression of genes involved in iron ion transport and homeostasis might be responsible for the increased tolerance to lignocellulosic inhibitors which cause marked oxidative stress (Moreno et al., 2022). Therefore, it is important to keep balance of iron inside mitochondria. According to the transcription profile of YRH396h-*ngg1*Δ compared to the control, genes involved in electron transport chain, ATPase activity, mitochondrion membrane, and matrix were all down-regulated. Moreover, oxidative stress tolerance ability of the *NGG1* deletion mutant was increased in 5 mM H_2_O_2_ (Figure 2). The above-mentioned results indicated that lower level of iron in engineered yeast strains carrying the XR-XDH pathway may be beneficial for the reduction of intracellular ROS level, improvement of XR activity, and weakening respiratory.

#### Differentially expressed genes related to nitrogen metabolism

##### Regulation of nitrogen metabolism

Nitrogen is one of the most important nutrients for cell growth, metabolism as well as various biological process. There is interplay between nitrogen and carbon regulation. Nitrogen transport and metabolic regulation may be both influenced by *NGG1* deficiency when performing xylose fermentation as revealed in our current study. It is known that preferred nitrogen sources could repress the utilization of non-preferred ones, and the bioprocess is termed nitrogen catabolite repression (NCR). There are mainly four TFs involved in regulating the expression of NCR-related genes, including activators Gln3p and Gat1p, and repressors Gzf3p and Dal80p (Zhang et al., 2018). As revealed by our current transcription data, *GAT1* was obviously up-regulated by 1.15-fold, and the repressor encoding gene *DAL80* was weakened by −0.5-fold. The variation might remodel the nitrogen utilization pattern for xylose utilization.

Furthermore, based on the potential regulatory network, *UGA3* and *STP1* are main regulators associated with the differentially transcribed genes affected by *NGG1* deletion (Supplementary Figure 5). *UGA3* is a TF involved in the induction of gamma-aminobutyrate (GABA) metabolism genes including *UGA1*, *UGA2*, and *UGA4*, which all take part in the utilization of GABA as a nitrogen source. The transcription level of *UGA1*, *UGA2*, and *UGA4* was improved by 2.55, 1.38, and 2.32-fold, respectively. *STP1* encodes a key effector of the Ssy1-Ptr3-Ssy5 (SPS) amino-acid-sensing pathway and plays an important role in amino acids uptake. As found in our data, transcriptional profile of amino acids metabolism was changed by *NGG1* deletion. The results here suggested that Ngg1p might be a regulator for nitrogen catabolism.

Another notable result is that 10 of the 24 amino acid transporters were differentially changed at transcription level by *NGG1* deletion, and most of these transporters exhibited improved transcription level (Table 1). The general amino acid permease (*GAP1*) which also works as a sensor for detecting the abundance of substrates was up-regulated significantly. Interestingly, transporters for preferred nitrogen resources (asparagine, glutamine, and arginine) and non-preferred amino acid (proline) showed enhanced expression simultaneously (Table 1). Permeases responsible for non-preferred nitrogen sources are repressed when preferred nitrogen sources are supplied for yeast cells (Zhang et al., 2018). Results of transcript levels here clearly indicated that the deficiency of *NGG1* in *S. cerevisiae* was associated with the transcription of genes involved in nitrogen transportation, NCR, and nitrogen metabolism systemically. Nitrogen level also has obvious influence on central carbon metabolism (He et al., 2018). Based on the simultaneous changed carbon and nitrogen metabolism, it can be reasonably presumed that *NGG1* is one of the core nodes for global regulation of both aspects in the process of xylose fermentation.

**TABLE 1:** Relative transcription levels of genes encoding amino acid transporters between YRH396h-*ngg1*Δ and YRH396h.

Gene	Substrate(s)	Log_2_ YRH396h-*ngg1*Δ/YRH396h ratio
*AGP1*	Asparagine, glutamine, other amino acids	1.07
*ALP1*	Arginine	1.58
*BAP3*	Cysteine, leucine, isoleucine, valine	3.34
*GAP1*	L-Amino acids	1.06
*PUT4*	Proline	1.67
*SAM3*	S-Adenosylmethionine	0.86
*UGA4*	γ-Aminobutyrate	2.32
*YCT1*	Cysteine	0.71
*MEP1*	Ammonium	–2.90
*MMP1*	S-Methylmethionine	–1.12

##### Metabolism of amino acids

In contrast to generally decreased transcription level of genes in central carbon metabolism such as TCA cycle, multiple genes for amino acid biosynthesis were upregulated in the *NGG1* deletion mutant, particularly histidine, cysteine, methionine, phenylalanine, and arginine (Figure 4). A significant alteration of transcription level in YRH396h-*ngg1*Δ was observed in the biosynthesis of histidine. The highest up-regulation gene was *HIS5*, which encodes a histidinol-phosphate aminotransferase. Within pathways of sulfur-amino acid biosynthesis, *MET17* and *MET6* were the most upregulated genes for O-acetyl homoserine-O-acetyl serine sulfhydrylase and methionine synthase. *MET17* and *MET6* are essential for methionine and cysteine biosynthesis, methionine biosynthesis and regeneration, respectively. Besides that, several genes involved in arginine biosynthesis were obviously affected in YRH396h-*ngg1*Δ when compared to YRH396h, such as the increased expression of *ARG1*, *ARG3*, *ARG8*, *CAR1*, and *ARG5*. According to the study of thermotolerant yeast *Kluyveromyces marxianus*, yeast cells depleted some amino acids biosynthetic pathways to conserve the precursor metabolites of carbon metabolism under the condition of high temperature (Marcišauskas et al., 2019). Recently, it was found that industrial thermotolerant *S. cerevisiae* Ethanol Red responds to high temperature by repression of proteins involved in arginine biosynthesis, including Arg1p, Arg5p, Arg6p, and Arg8p (Pinheiro et al., 2020). We assume that higher expression of genes for arginine biosynthesis and global regulation of amino acid metabolic pathways might be a reason for decreased thermotolerance of *NGG1* deletion mutant as shown in Figure 2.

**FIGURE 5:**
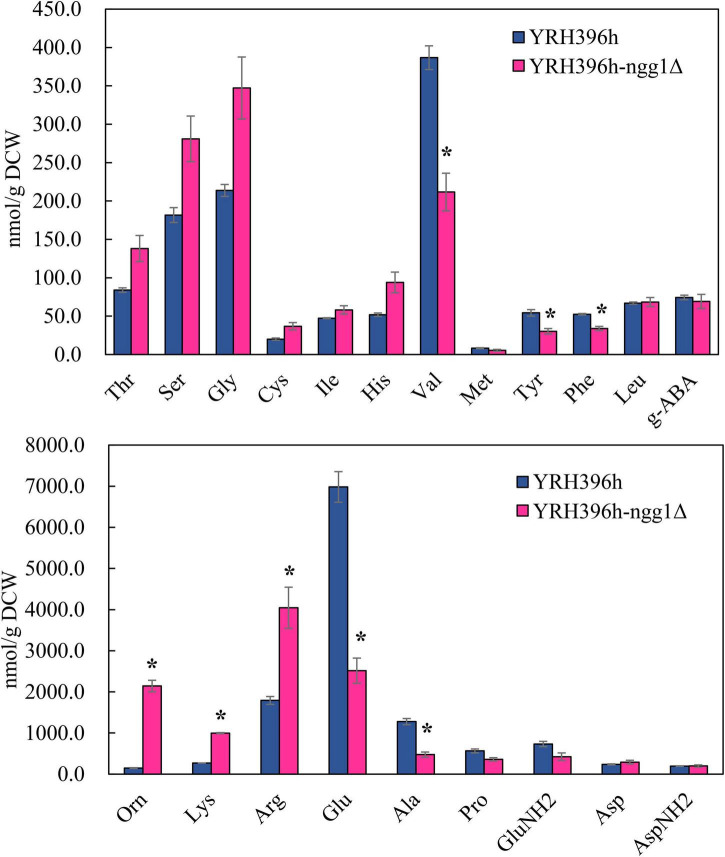
Impact of *NGG1* deletion on the content of intracellular amino acids of YRH396h. The *NGG1* deletion strain and its parent strain *S. cerevisiae* YRH396h were grown in YP liquid medium using xylose as the sole carbon source, and cells were collected at log phase for the analysis of intracellular amino acids. *P < 0.05.

Subsequently, the content of intracellular amino acids of YRH396h-*ngg1*Δ and YRH396h was measured under the condition of xylose fermentation (Figure 5). Consistent with the results of comparative transcriptome analysis, the content of arginine in YRH396h-*ngg1*Δ was 4,044.3 nmol/g DCW, higher than that in YRH396h (1,792.5 nmol/g DCW). Interestingly, significantly lower contents of valine, tryptophan, glutamate, and alanine, but higher level of ornithine was detected when compared to the *NGG1* deletion mutant with the control strain, albeit no difference in the transcriptomic data was observed. Transcription of genes involved in aromatic amino acids biosynthesis, especially for phenylalanine, were upregulated, such as *ARO2*, *ARO7*, and *PHA2*. However, the phenylalanine content in the YRH396h-*ngg1*Δ was 35.24% lower than that of the control. In contrast, although the transcriptional levels of lysine biosynthesis-related genes (*LYS4*, *LYS12*, and *LYS9*) were decreased by −0.79, −1.06, and −1.11-fold, respectively, its content was increased obviously in the cells of YRH396h-*ngg1*Δ (Figure 5A). These results further supported the notion that *NGG1* regulates amino acids biosynthesis during xylose metabolism, and the accumulation of amino acids may be regulated at additional levels in addition to biosynthesis, because the uptake of the amino acids as well as conversion of them into other metabolites may also be affected, which warrant further investigation. The varied expression levels of transporters including *AGP1*, *ALP1*, *BAP3*, *GAP1*, *PUT4*, and *YCT1*, which are functional in amino acid transport, were possibly related to the accumulation of intracellular amino acids. For instance, higher content of arginine might be due to the simultaneously enhanced transcription of arginine transporter encoding gene *ALP1* by 1.58-fold as well as these genes involved in arginine biosynthesis.

Previously, it was inferred that cellular amino acid metabolic process was possibly involved in the transcription regulation of xylose metabolism by *S. cerevisiae*, regardless of host genetic background and xylose pathway (Feng and Zhao, 2013a). Recently, through transcriptional analysis, it was conjectured that improved amino acids biosynthesis was related to a better xylose catabolism by *S. cerevisiae*, and the lag phase in xylose was shortened by overexpression of *GCN4*, which is a global transcription activator for amino acids biosynthesis genes (Li et al., 2021). In this study, although the transcription level of *GCN4* was unchanged, it was found that Gcn4p was an enriched TF to regulate the differentially transcribed genes in response to the deficiency of *NGG1* (Supplementary Figure 3). Our results indicated that Ngg1p might be a global regulator of amino acids metabolism in addition to its known function in glucose repression.

It has reported that biomass accumulations and ethanol production of *Pichia stipitis* were influenced by the presence of amino acids. It has confirmed that arginine had a positive impact on the growth of *P. stipitis*, while tryptophan, glutamate, and alanine had a negative impact (Slininger et al., 2006). Interestingly, the obviously increased content of arginine and decrease of tryptophan, glutamate, and alanine were revealed in YRH396h-*ngg1*Δ (Figure 5). This suggested that the variation of intracellular amino acid contents of YRH396h-*ngg1*Δ was beneficial for its biomass accumulation. Here, we speculated that Ngg1p was a key transcription activator to regulate xylose metabolism and biomass accumulation of *S. cerevisiae* through the regulation of amino acids biosynthesis. It will be interesting to further study the genetic characteristics of YB-2625 and explore innate regulatory network of *NGG1* with other key TFs to reveal the underlying mechanism. In addition, more detailed transcriptomics and metabolomics studies will be performed to reveal the connections between gene expressions and metabolism affected by *NGG1*.

**FIGURE 6:**
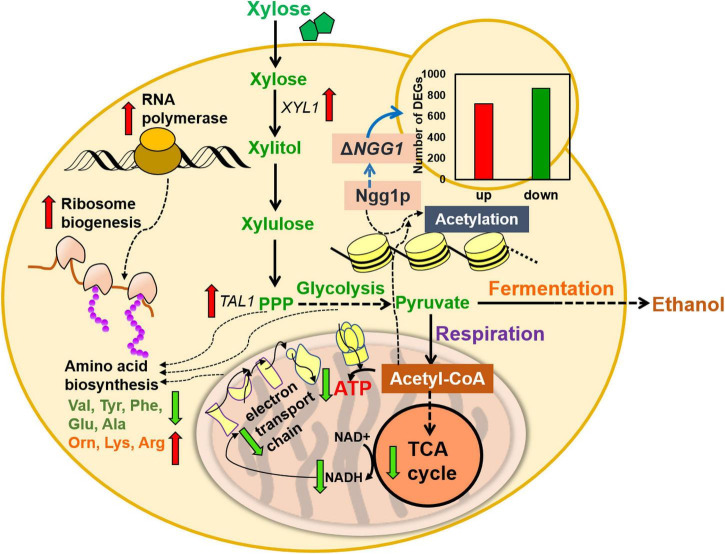
Proposed model for improved xylose metabolism by *NGG1* deletion in *S. cerevisiae* YRH396h. The down-regulated genes primarily participated in mitochondrial function, TCA cycle, ATP biosynthesis, respiration, as well as NADH generation. The *NGG1* deletion mutant also showed different content of intracellular amino acids compared to the control strain.

In summary, global transcription analysis revealed weakened expression of genes related to mitochondrion function, such as TCA cycle, electron transport chain, and ATPase activity by *NGG1* deletion. In addition, multiple genes involved in amino acid transportation and biosynthesis were upregulated. The obviously lower expression of gene encoding acetyl coenzyme A synthase was caused by *NGG1* deletion. Furthermore, Ngg1p absence down-regulated pathway genes involved in respiration, ATP biosynthesis, and TCA cycle (Figure 6). Xylose fermentation rather than respiration was stimulated by comprehensive change of these genes. Therefore, the *NGG1* deletion mutant tend to recognize xylose as a fermentative carbon source, and the impaired respiration might contribute to the higher ethanol accumulation. Additionally, iron homeostasis which is related to ROS accumulation was impacted by *NGG1* deficiency. In accordance with down-regulated genes of ROS production, the *NGG1* deletion mutant exhibited better performance under oxidative stress, which also had positive correlations with the superior xylose metabolism ability. On the other hand, significantly varied terms were concentrated on nitrogen transportation, regulation, and amino acid metabolism. It has confirmed that appropriate amino acid metabolic process was conducive to xylose metabolism. Obvious change of arginine, tryptophan, glutamate, and alanine was revealed by *NGG1* deletion. We hypothesized that this variation might have a beneficial effect in biomass accumulations and ethanol production of xylose-fermenting yeast. Altogether, the analysis demonstrated that cellular metabolic remodeling occurred when deleting *NGG1* in yeast cells. The global rewiring of pathways involved in respiration, TCA cycle, iron homeostasis, and amino acid biosynthesis might serve as key factors for enhanced xylose fermentation of *NGG1* deletion mutant. In future study, it will be interesting to detect the influence of single copy or complete deletion of *NGG1* in diploid strains and then compare the changes of metabolic network. In order to develop more robust yeast for lignocellulosic ethanol production, fine-graded regulation of *NGG1* to improve stress tolerance and xylose metabolism simultaneously could be carried out in future study.

## Conclusion

Deletion of *NGG1* improved growth and ethanol fermentation in the presence of xylose. Comparative transcriptome analysis implied that Ngg1p is a key regulator of nitrogen and carbon metabolism, and we further confirmed that the accumulation of intracellular amino acids was regulated by *NGG1*. Our studies provide insights into the function of Ngg1p in cell metabolism, and also basis for further manipulation of *NGG1* as a novel target to construct more efficient strains for xylose utilization.

## Data availability statement

The original contributions presented in this study are included in the article/Supplementary material, further inquiries can be directed to the corresponding author/s.

## Author contributions

CC and X-QZ designed the experiments. CC, W-BW, and LB performed the experiments as well as data analysis. CC, M-LS, and R-QT prepared the draft of the manuscript. HA and X-QZ contributed to manuscript preparation and revision. All authors read and approved the final manuscript.

## Footnotes

1
http://www.geneontology.org/

2
http://www.yeastract.com/
