# Supplementary material for: Deletion of NGG1 in a recombinant Saccharomyces cerevisiae improved xylose utilization and affected transcription of genes related to amino acid metabolism
Source: Front Microbiol. 2022 Sep 8;13:960114. doi: 10.3389/fmicb.2022.960114 (PMC9493327; doi:10.3389/fmicb.2022.960114)
Supplement: Supplementary file 1 [file Data_Sheet_1.pdf]

Table S1 Yeast strains used in this study

Yeast strain	Genotype	Source
S288c	*S. cerevisiae*, laboratory strain	Stored in our lab
YB-2625	*S. cerevisiae* isolated from bagasse	NRRL
YRH396	YB-2625 *ho*Δ*::*(KanMX4; P_{PGK1}-*XYL1*-T_{PGK1}; P_{ADH1}-*XYL2*-T_{ADH1}; P_{HXT7}-*XKS1*-T_{HXT7})	(Hector et al., 2011)
YRH396h	Haploid of YRH396	This work
YRH396h-*ngg1*Δ	YRH396h *ngg1*Δ*::*HphMX	This work

Table S2 Primers used in this study for gene manipulation and verification

Primer name	Sequence 5’-3’
*ngg1*Δ-F	ATGCCTAGACATGGAAGAAGAGGAAAACTGCCAAAAGGCGCAGCGACATGGAGGCCCAG
*ngg1*Δ-R	TTAATTTAGTTCCACGTCCTTATTAGTGTTTTGTGCAAATCGTCCCAAAACCTTCTCAA
hph-in-R	TGGTCAAGACCAATGCGGAG
NGG1-F	ATGCCTAGACATGGAAG

The underlined sequence was homologous arm of HphMX marker.

Table S3 Primers for real-time quantitative PCR analysis

Primer name	Sequence 5'-3'
RT-*XYL1*-F	GAGGTGCTACCATCAAGCCATCTG
RT-*XYL1*-R	CGAACGAAGAGTAAGCGGTGACAG
RT-*XYL2*-F	TCGCCGCTACTCCTAACTCCAAG
RT-*XYL2*-R	TCAACAAGAGCACCGAGTTCCAAG
RT-*XKS1*-F	AGCACGGGTCTGTCTACTGG
RT-*XKS1*-R	AGGCCCACCTATGCACTCTT
RT-*SOD1*-F	TGTCTCTGCTGGTCCTCACTTC
RT-*SOD1*-R	CACACCATTTTCGTCCGTCTT
RT-*PRX1*-F	TCGACGCCTTGCAATTGACTGAC
RT-*PRX1*-R	GCCTTCGCCTCATCATTGGAGAC
RT-*CTT1*-F	CAATTGCCCGTCAACAGA
RT-*CTT1*-R	ATTTGGCTCAGGACCGAA
RT-*ASK10*-F	ATGACGCCTCCTCCATCCACTC
RT-*ASK10*-R	TCCGTAGTAGAGGTTGGCTGAGC

Table S4 Comparison of fold changes of selected genes between RNA-seq and qPCR analyses

Gene	RT-qPCR analysis	RNA-seq analysis
*XYL1**	1.90	ND
*XYL2**	1.11	ND
XKS1	0.49	0.45
SOD1	1.25	ND
PRX1	0.59	0.46
CTT1	1.89	1.72
ASK10	0.67	0.56

* These two genes are not present in the genome of S288c. The qPCR and RNA-seq results were all calculated by the $2^{-\Delta\Delta Ct}$ method. ND: No significant difference of this gene was found in the RNA-seq analysis between YRH396h-*ngg1*Δ and YRH396h.

Table S5 Fold change of xylose metabolism relative genes between YRH396h-*ngg1*Δ and YRH396h

Gene	Log_2 YRH396h-*ngg1*Δ/YRH396h ratio
ASK10	-0.84
HSP26	ND
SSA1	ND
HSP104	ND
HOG1	ND
GRE3	ND
IRA2	-0.80
ISU1	-0.83
SSK2	-0.52
CAT8	-0.93
HAP4	ND
ADR1	-1.11
MSN2	ND
MSN4	0.67
GIS1	-1.03
AFT2	ND
USV1	-0.53
SNF1	ND

ND: No significant difference of this gene was found in the RNA-seq analysis between YRH396h-*ngg1*Δ and YRH396h.

Figure legends for the supplementary data

Figure S1 Changed lysine acetylation level of YRH396h exerted by *NGG1* deficiency. The cell extracts from YRH396h-*ngg1*Δ and YRH396h grown in YPX medium at log phase were separated by SDS-PAGE respectively. The samples were determined by western blot using anti-acetyllysine antibody (1:1000) as the primary antibody and goat anti-mouse IgG (1:5000) as the 2nd antibody. The arrows indicate different immunoblot signals between the two strains.

Figure S2. Growth ability of different haploids derived from YRH396 in xylose or mixed-sugar. The sixteen haploid isolates (bars No. 1-16) and diploid YRH396 (bar No. 0) were cultivated in 48 deep-well baffled plates by 10% of inoculation in 1 mL YPX40 (A) or YPD80X40 (B) medium to determine the growth ability. The mixed-sugar medium (YPD80X40) composed of 4 g/L yeast exact, 3 g/L peptone, 80 g/L glucose, and 40 g/L xylose, while 40 g/L xylose was used as sole carbon source in YPX40 medium. After cultured at 220 rpm and 30°C for 96 h, the cell growth ability was determined by OD_{600}. The orange bar stand for YRH396h.

Figure S3. Top 20 enriched transcription factors regulating the significantly changed genes between the transcriptomic profile of YRH396h-*ngg1*Δ and the control strain YRH396h during xylose fermentation. Enriched transcription factors were analyzed with differentially expressed genes (Log_2 ratio ≥ 0.5 or ≤ -0.5, FDR ≤ 0.001). Yeast strain with *NGG1* deletion and its parent strain *S. cerevisiae* YRH396h were grown in YPX liquid medium using xylose as the sole carbon source, and culture broth was collected at the designated time point. The value of y-axis indicated the percentage of changed genes regulated by transcription factors.

Figure S4. Enriched categories of obviously down-regulated genes between YRH396h-*ngg1*Δ and the control strain YRH396h. Gene Ontology (GO) enrichment was analyzed with differentially expressed genes (Log_2 ratio ≤ -0.5, FDR ≤ 0.001). **(A) Molecular Function (MF)-**1. oxidoreductase

activity, 2. catalytic activity, 3. glutathione transferase activity, 4. peroxidase activity, 5. succinate dehydrogenase activity, 6. glutathione peroxidase activity, 7. electron carrier activity, 8. proton-transporting ATPase activity, 9. transporter activity, 10. metal ion binding; **(B) Biological Process (BP)-**1. oxidation-reduction process, 2. tricarboxylic acid cycle, 3. ATP biosynthetic process, 4. electron transport chain, 5. ergosterol biosynthetic process, 6. cellular carbohydrate metabolic process, 7. ion transport, 8. NADPH regeneration, 9. cellular response to oxidative stress, 10. carbohydrate metabolic process; **(C) Cellular Component (CC) -**1. mitochondrion, 2. membrane, 3. integral to membrane, 4. mitochondrial nucleoid, 5. plasma membrane, 6. mitochondrial inner membrane, 7. ATP synthase complex, 8. mitochondrial matrix, 9. mitochondrial intermembrane space, 10. respiratory chain.

Figure S5. Regulators and target genes of *NGG1*. The interaction network was obtained by SGD. Regulators of *NGG1* are marked in green, and purple nodes stand for target genes of *NGG1*.

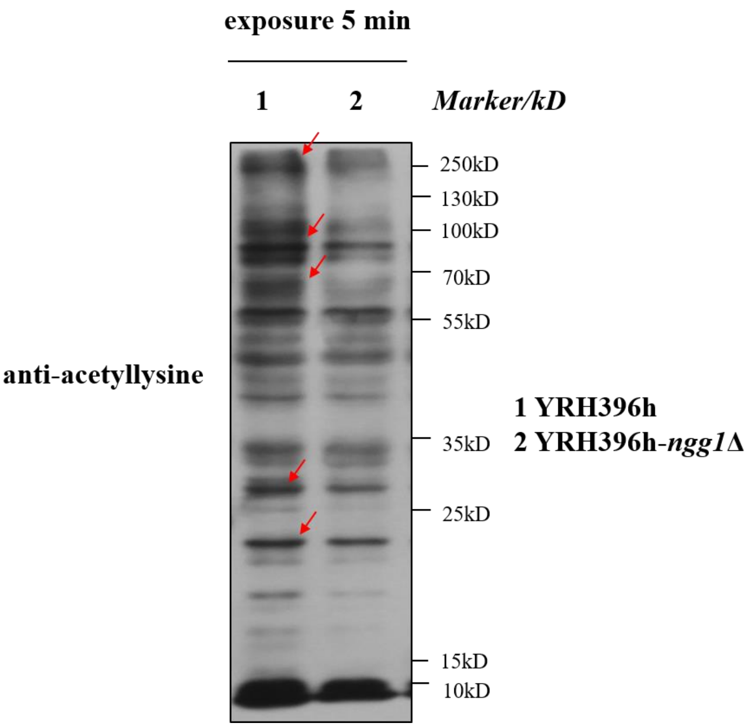
exposure 5 min
1
2
Marker/kD
250kD
130kD
100kD
70kD
55kD
anti-acetyllysine
1 YRH396h
2 YRH396h-ngg1Δ
35kD
25kD
15kD
10kD

Fig. S1

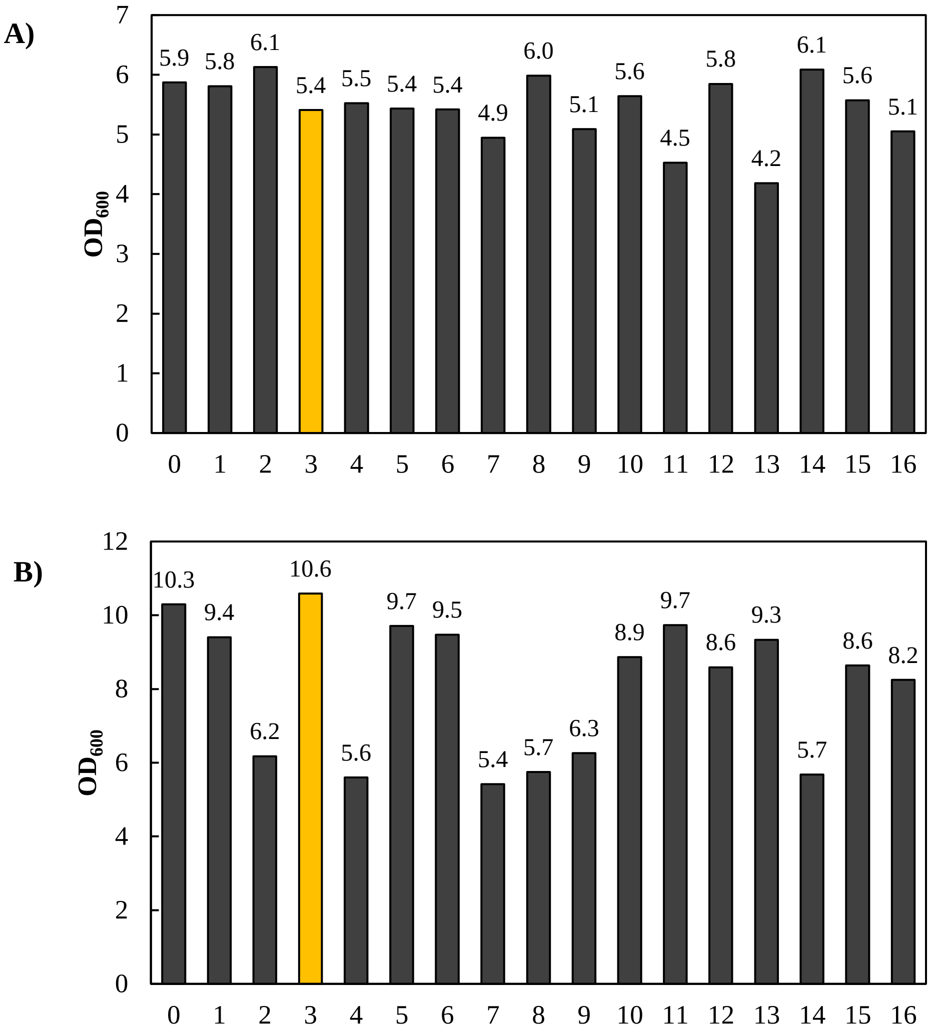
A)
OD600
0
1
2
3
4
5
6
7
5.9 5.8 6.1 5.4 5.5 5.4 5.4 4.9 6.0 5.1 5.6 4.5 5.8 4.2 6.1 5.6 5.1
0 1 2 3 4 5 6 7 8 9 10 11 12 13 14 15 16
B)
OD600
0
2
4
6
8
10
12
10.3 9.4 6.2 10.6 5.6 9.7 9.5 5.4 5.7 6.3 8.9 9.7 8.6 9.3 5.7 8.6 8.2
0 1 2 3 4 5 6 7 8 9 10 11 12 13 14 15 16

Fig. S2

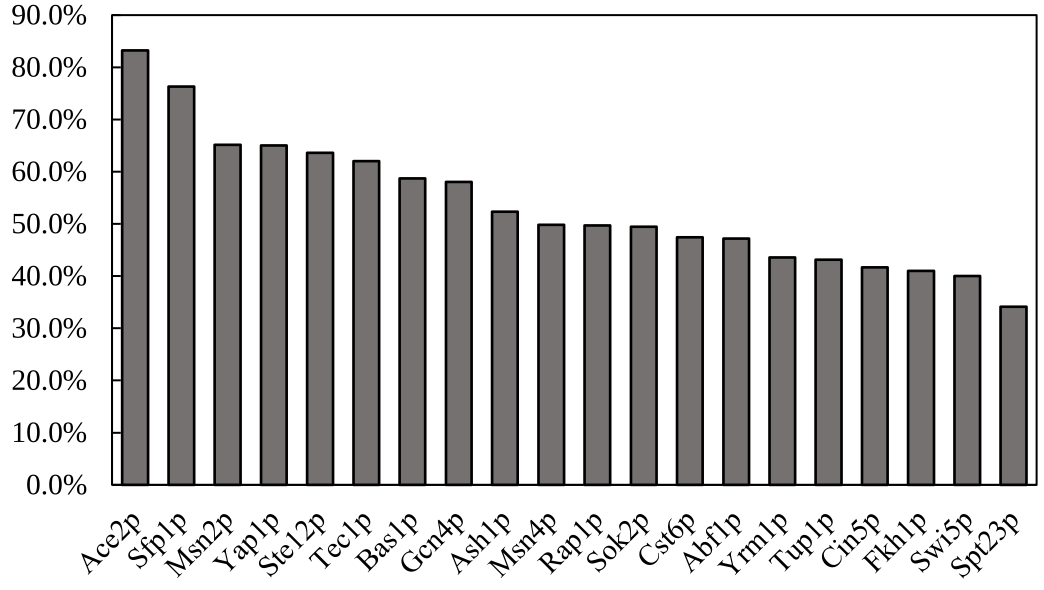
90.0%
80.0%
70.0%
60.0%
50.0%
40.0%
30.0%
20.0%
10.0%
0.0%
Ace2p
Sfp1p
Msn2p
Yap1p
Ste12p
Tec1p
Bas1p
Gcn4p
Ash1p
Msn4p
Rap1p
Sok2p
Cst6p
Abf1p
Yrm1p
Tup1p
Cin5p
Fkh1p
Swi5p
Spt23p

Fig. S3

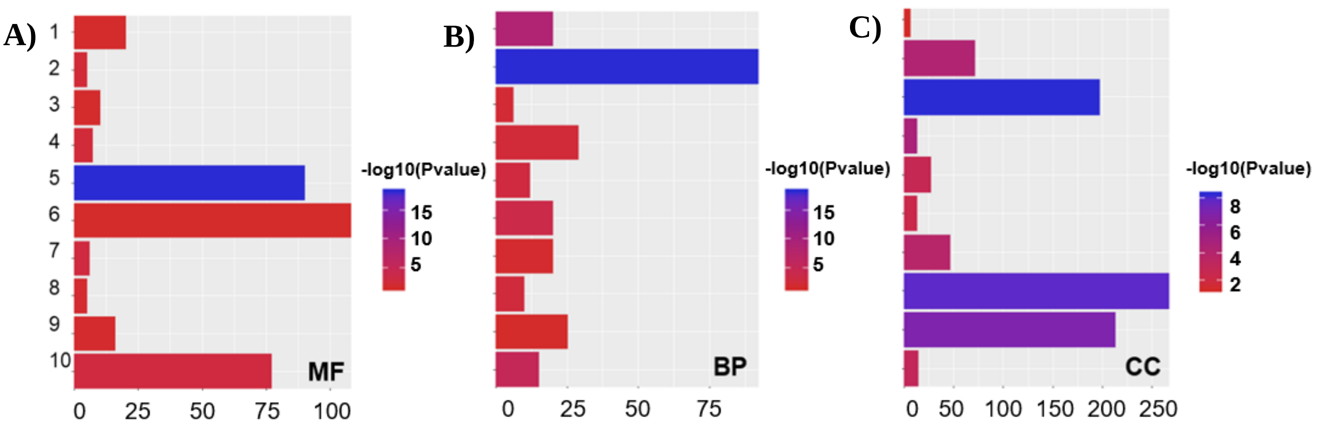
A)
B)
C)
1
2
3
4
5
6
7
8
9
10
MF
BP
CC
-log10(Pvalue)
15
10
5
8
6
4
2
0
25
50
75
100
150
200
250

Fig. S4

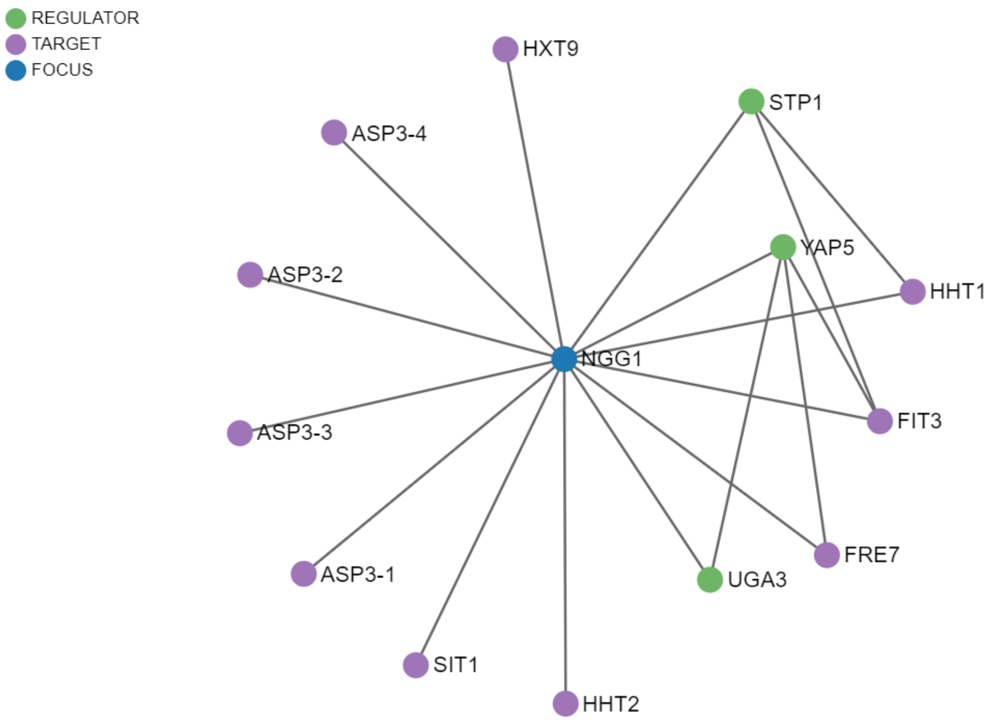
REGULATOR
TARGET
FOCUS
HXT9
STP1
ASP3-4
YAP5
ASP3-2
HHT1
NGG1
FIT3
ASP3-3
FRE7
ASP3-1
UGA3
SIT1
HHT2

Fig. S5
